# The role of sleep in Alzheimer’s disease: a mini review

**DOI:** 10.3389/fnins.2025.1428733

**Published:** 2025-02-05

**Authors:** Jay Pathmanathan, M. Brandon Westover, Sudhir Sivakumaran, Jacob Donoghue, Corey B. Puryear

**Affiliations:** Beacon Biosignals, Boston, MA, United States

**Keywords:** sleep, Alzheimer’s disease, review, sleep monitoring, neurodegenerative disease

## Abstract

Sleep is a stereotyped and well-preserved series of neurophysiological states that are essential for overall health and brain functioning. Emerging research suggests that sleep disturbances are not only associated with but also causally contribute to neurodegenerative disease onset and progression. This mini-review examines some of the current knowledge and evidence for relationships between sleep abnormalities and Alzheimer’s disease within context of possible uses and limitations of sleep biomarkers for evaluation of Alzheimer’s disease. Understanding these relationships could lead to readily accessible and easily quantifiable biomarkers of Alzheimer’s dementia.

## Introduction

Sleep is an evolutionarily preserved and necessary biological process whose purpose remains poorly understood (Cirelli and Tononi, 2008). Nevertheless, the importance of sleep in cognitive functioning has been demonstrated through studies on the impacts of sleep deprivation on cognition (Durmer, 2005; Alhola and Polo-Kantola, 2007; Csipo et al., 2021) and correlations between sleep physiology and cognitive performance. Alterations in sleep macro-architecture, such as time in rapid eye-movement (REM) sleep, has been linked to worsening cognitive performance with age (Song et al., 2015). Sleep micro-architecture has been hypothesized to play a role in both network optimization and metabolic activities necessary for network maintenance. For example, sleep spindle properties have been linked to memory consolidation (Schabus et al., 2004; Fogel and Smith, 2011; Rasch and Born, 2013; Latchoumane et al., 2017; Fernandez and Lüthi, 2020; Adra et al., 2022), slow-wave delta power has been linked to synaptic plasticity (Tononi, 2009; Miyamoto et al., 2017; Timofeev and Chauvette, 2017), and sleep duration—particularly slow-wave sleep (SWS) time—has been shown to be associated with glymphatic clearance of waste metabolites and toxic protein breakdown products (including the toxic protein fragments involved in neurodegenerative processes; Xie et al., 2013; Reddy and Van Der Werf, 2020; Buongiorno et al., 2023; Voumvourakis et al., 2023).

In healthy aging, known changes to sleep include a circadian shift to earlier sleep, increased arousals during sleep resulting in more light sleep stages (N1 and N2) at the cost of reduced deep sleep (i.e., slow wave sleep, SWS) and REM sleep, more daytime naps, decreased sleep efficiency with greater wake after sleep onset, and an increasing incidence of insomnia (Li et al., 2018). Furthermore, aging results in micro-architectural changes including reduced spindle density, spindle coherence, and SWS delta power (Yokoyama et al., 1996; Purcell et al., 2017; Palepu et al., 2023). Many of these macro-and micro-architectural features have been correlated with impaired cognition, brain network dysfunction, and reduction in clearance of toxic metabolites (Tononi and Cirelli, 2003, 2006; Xie et al., 2013; Ju, 2017).

When and why are sleep changes pathological and predictive of neurodegenerative disease, and when are they simply a normal part of aging? Is there evidence that sleep pathology contributes to the development of neurodegenerative dementia? Does sleep pathology, either independent of disease or because of the degeneration of cerebral sleep networks, contribute to the exacerbation or acceleration of an underlying neurodegenerative process? This review examines our understanding of potentially bi-directional relationships between sleep pathology and Alzheimer’s Dementia, necessary future avenues of research, and the potential value and roles of quantitative sleep metrics as biomarkers of brain health.

## Links between Alzheimer’s disease and sleep pathology

Alzheimer’s Disease (AD) is a disease of progressive neurodegeneration related to the accumulation of toxic beta-amyloid and, eventually, phosphorylated tau protein fragments (Knopman et al., 2021). These insoluble collections are thought to result from the self-aggregating nature of certain amyloid and tau breakdown fragments that result from the abnormal cleavage of ubiquitously present amyloid precursor and tau proteins. Sleep is a natural target of research given its role in both the rate of accumulation and rate of removal of such toxic protein species (Xie et al., 2013; Winer et al., 2019; Sadeghmousavi et al., 2020; Winer et al., 2020b).

Generation of these breakdown products are tied to neuronal activity, which is highest during wakeful periods (Cirrito et al., 2005; Kang et al., 2009; Lucey et al., 2018). Therefore loss of sleep, as occurs in insomnia or states of hypervigilance, should naturally result in greater production of potentially toxic metabolic waste products (Lucey et al., 2018). Conversely, sleep is associated with more active glymphatic clearance of metabolic waste, particularly during slow wave sleep (Reddy and Van Der Werf, 2020; Wafford, 2021; Bohr et al., 2022; Voumvourakis et al., 2023).

These findings should be observable given the high frequency of sleep disorders and Alzheimer’s disease. If sleep pathology is truly linked to development or progression of Alzheimer’s disease then patients with sleep disorders should have a higher risk and/or more rapid disease course. Multiple studies have indeed found a strong association between sleep disorders like insomnia (Sadeghmousavi et al., 2020; Benca et al., 2022), sleep apnea (Ancoli-Israel et al., 1991; Emamian et al., 2016; Andrade et al., 2018; Sharma et al., 2018), and circadian rhythm disorders (Homolak et al., 2018; Ahmad et al., 2023) and increased risk of developing Alzheimer’s disease. Clear associations have been described between sleep macro-and micro-architectural disturbances and development of Alzheimer’s pathology. Disturbances in total sleep time, sleep efficiency (percent of attempted sleep time spent asleep), sleep latency, and slow-wave activity (SWA, amount or power of 0–4 Hz activity in slow-wave sleep or overall). Changes in these metrics, particularly SWA and sleep efficiency, have been reported years prior to clinical presentation (Lucey et al., 2019; Lee et al., 2020; Winer et al., 2020b; Sabia et al., 2021; Benca et al., 2022). While these findings remain preliminary, sleep poses an opportune target of study in Alzheimer’s due to the low cost of evaluation, ability to intervene on many sleep disturbances, and hypothesized potential for disease prevention (as opposed to treatment).

## Sleep macroarchitecture and Alzheimer’s disease

Sleep is series of regulated brain states that present with stereotyped physiological characteristics, classified originally based on patterns of brain activity recorded using electroencephalography (EEG) into N1 (drowsiness), N2, slow wave sleep (SWS, formerly N3 and N4), and rapid eye movement (REM) sleep. Stages N1, N2, and SWS are together considered non-REM (NREM) sleep. These stages can be distinguished easily based on EEG, but can also be assessed with more moderate sensitivity and specificity with peripheral monitoring of respiration, pulse, EKG, and or actigraphy/movement (Green et al., 2022). Collectively, sleep stage information and data directly derived from these metrics are considered sleep “macroquantities.” These include (but are not limited to) time in each stage, total sleep time (TST), sleep onset latency (SOL, time from attempting to sleep to the first sleep state), sleep efficiency (SE, percent of attempted sleep with actual sleep), wake after sleep onset (WASO), and number of awakenings. Patient self-reported sleep diaries can also be used to estimate total sleep time, wake after sleep onset, and number of awakenings, though diaries and surveys have lower concordance with gold standard polysomnography (PSG) than actigraphy (Lehrer et al., 2022).

The hypothesis that sleep plays a role in the development of AD arises from observations of the role of sleep in inflammatory cycles, metabolic waste clearance, and memory consolidation (Sadeghmousavi et al., 2020)—with impairments leading to an environment more conducive to aggregation of toxic protein aggregates. Several large retrospective studies have examined the relationship between macroarchitectural sleep disturbances and development of AD. Winer et al. (2020a) used EEG based polysomnography to characterize sleep in 32 cognitively normal adults, and then them with serial [11C] PiB PET scans to monitor amyloid-beta accumulation. They found that SE and TST were inversely correlated with accumulation of toxic protein aggregates linked to AD, albeit not a formal diagnosis of AD. These findings suggest sleep disorders would be more prevalent in patients with AD, but also that sleep disorders would be more common in pre-symptomatic (healthy) people who later develop AD.

Benca et al. (2022) conducted a meta-analysis of 58 studies examining insomnia (a disorder involving difficulty initiating or maintaining sleep—including *perceived* problems with TST, SE, SOL, and WASO) in patients with clinically suspected AD of varying severity. Reported sleep disturbances were clearly associated with poor health status and reduced quality of life based on patient reported outcomes. Poor sleep also was also linked to anxiety, increased burden for caregivers, and was associated with a higher likelihood of admission to long-term care facilities. Data was not available to demonstrate that pharmacological interventions on sleep disturbances impacted AD progression. Furthermore, sleep was not necessarily quantitatively assessed (a technically challenging endeavor in large patient populations, given the difficulty in obtaining brain state).

Large studies examining sleep in pre-symptomatic patients have also demonstrated links between sleep microquantities and development of AD. Lysen et al. (2020) followed thousands of subjects in a prospective observational cohort study on health. 2063 participants (mean age of 62) underwent 7 days of wrist actigraphy and maintained sleep diaries. After exclusions (those with cognitive dysfunction, whose actigraphy device malfunctioned, who failed to collect at least 4 days of data, or who were < 55 years old at baseline), they followed 1,322 subjects until a diagnosis of dementia, death or loss to follow-up, or the year 2016 (8.8 years on average). 49 subjects were eventually diagnosed with AD, and these subjects had lower SE, higher WASO, longer time in bed, and longer SOL (each of these sleep features demonstrating a statistically significantly higher hazard ratio for development of AD). However, neither 24-h activity patterns nor TST were associated with subsequent risk of AD (which is notably disparate from other reports described below). Furthermore, these findings were based only on 1 week of sleep assessment performed years prior to ultimate diagnosis. In this population, it is uncertain if sleep disturbances were transient or maintained, and what impact this would have on dementia risk.

In a large observational cohort study of 7,959 participants followed since the late 1980s (Whitehall II study, following 10,308 British civil servants and collecting a range of clinical metrics and health records) sleep duration was measured using questionnaires administered every 5–10 years (Sabia et al., 2021). Among participants in their 50s, both self-reported short and long TST (with 7 h being taken as the normal reference) were significantly associated with a higher risk of subsequent dementia. For participants in their 60s and 70s, only short TST (<7 h) was significantly associated with subsequent development of dementia. However, note that Winer et al. found a similar relationship between short TST and worse cognitive performance (not necessarily development of AD specifically though), but also found worse cognitive performance with TST longer than 8 h (a “U-shaped association”) when examining 4,417 cognitively normal participants with a mean age in their early 70s from 4 countries (Winer et al., 2021). In the Whitehall II study, 3,888 subjects aged 60–83, also underwent a single period of actigraphy based sleep assessment to provide quantitative TST, which demonstrated that the hazard ratio for developing dementia monotonously increased for both short and long sleep time (duration of sleep less or more than 7 h was directly proportional to dementia risk, with a maximum hazard ratio of over 3.5x at 4 h of sleep). In this study, dementia could be reliably determined through national healthcare databases, but not precise dementia type. Therefore, the risk of developing Alzheimer’s specifically is not known from this study (though AD generally accounts for the majority of dementia cases).

Wong and Lovier examined longitudinal self-reported sleep data from 6,284 United States Medicare beneficiaries age 65 years and older who participated in the National Health and Aging Trends Study and were cognitively normal in 2011 (Wong and Lovier, 2023). Eventual AD diagnosis was based on cognitive testing results combined with self-report or physician report of Alzheimer’s type dementia. In such large studies, it is important to note the difficulty in establishing an Alzheimer’s Diagnosis and the resulting diagnostic error from this complexity. Importantly, the diagnostic fidelity of this large cohort has been examined and found to be 66% sensitive and 87% specific (as compared to the Aging, Demographics, and Memory Study, in which diagnosis was determined by expert consensus adjudication of diagnosis, for a subset of participants who were enrolled in both studies). These authors found that sleep-initiation insomnia (involving prolonged sleep onset latency) was significantly associated with subsequent AD (hazard ratio 1.5x), but sleep maintenance insomnia (problems with WASO and SE) was not. Interestingly, sleep maintenance insomnia had a lower hazard ratio of 0.66x (problems maintaining sleep appeared to be protective in this study). It should also be noted that models of sleep initiation insomnia adjusted for sociodemographic status were no longer statistically significant, although these adjusted models demonstrated that use of sleep medication was significantly associated with a 30% increased risk of AD.

Taken together, these large studies demonstrate a strong association between disturbances in sleep macroquantities (particularly those associated with insomnia) and a subsequent diagnosis of dementia. However, there is variability in findings and there are inconsistencies in the value of specific sleep metrics. A number of factors limit prior research, including the complexity of diagnosing AD, the complexity of accurately quantifying sleep macroquantities, and inaccuracies in subjective self-reports of sleep. AD diagnoses based solely on clinical criteria may not be accurate (many large studies provide no estimate of diagnostic accuracy), but the high cost and low availability of AD diagnostic tests with high sensitivity and specificity (as outlined below) result in limited availability. Similarly, EEG based sleep measurement, which is the gold standard for quantification of sleep, is difficult to obtain over multiple time points and in patients without profound sleep pathology due to limited ambulatory/at-home hardware options and high costs of in-lab assessment. Green et al. evaluated multiple non-invasive wearable sleep-measuring devices in older adults (Green et al., 2022) and found that TST and SE were consistently overestimated, while SWS and REM measurements were inaccurate in all devices except one EEG-based headband device (which was itself limited by data quality issues). Next generation EEG-based sleep monitoring hardware devices are becoming available and may improve quantification of sleep macro-architecture.

## Sleep microarchitecture

In addition to sleep macroarchitecture, brain activity within sleep stages has also been associated with cognition and memory. For example, sleep spindle patterns seen in N2 and SWS and power and frequency of slow waves in SWS have been implicated in memory consolidation, synaptic remodeling, and clearance of toxic metabolites (Rasch and Born, 2013). These within-stage EEG features, sleep microarchitecture, have therefore been of interest as diagnostic and therapeutic biomarkers of AD.

Prior studies have also shown that glymphatic clearance of toxic metabolites is maximal in SWS and is associated with clearance of soluble amyloid-beta fragments (Iliff et al., 2012; Ju, 2017; Jiang-Xie et al., 2024). Furthermore, a study of 8 human participants with indwelling lumbar spinal catheters demonstrated that one night of sleep deprivation resulted in a 30% increase in soluble amyloid species in CSF vs. those who slept normally or were given the sleep promoting drug, sodium oxybate (Lucey et al., 2018). Winer et al. (2019, 2020a) evaluated 31 cognitively normal older participants with EEG based sleep staging followed by 18F-FTP and PIB PET scans to quantify tau and amyloid within the brain. They found that tighter coupling between slow oscillations and spindles, which may reflect hippocampal-neocortical communication in memory consolidation (Muehlroth et al., 2019), was inversely correlated with both tau and amyloid levels as measured by PET (weaker coupling was associated with higher protein abnormality within the mesial temporal lobe). In contrast, the amount of very low frequency (<1 Hz) brain slow wave activity (SWA) during NREM sleep, which has been linked to regulation of synaptic strength (Tononi and Cirelli, 2006; Tononi, 2009), glymphatic clearance of metabolic waste (Iliff et al., 2012; Yi et al., 2022), and immune regulation (Lange et al., 2011; Besedovsky et al., 2012), was inversely correlated with buildup of amyloid-beta over subsequent years (Winer et al., 2020b). However, while Winer et al. did not find an increase in tau with reduced SWA, Lucey and colleagues analyzed CSF samples from their amyloid csf study and found that 1 night of sleep deprivation in healthy adults increased CSF tau by 50% (Lucey et al., 2019).

Despite clear evidence that sleep microarchitecture impacts the factors believed to lead to AD, to date there has not been a study demonstrating the link between sleep microarchitecture and AD diagnosis. This is due, at least in part, to the long time course of the disease, where sleep pathology could contribute to a diagnosis years or decades later. Nevertheless, the link between sleep pathology and Alzheimer pathology precursor proteins is increasingly compelling and will be the target of future research.

## Comparison to other biomarkers

Sleep pathology could provide intervenable biomarkers of AD, but these biomarkers will not serve the same role as traditional biomarkers. Early AD diagnoses are most commonly made on purely clinical grounds or with additional ancillary testing including MRI imaging, neuropsychological testing, CSF analysis for amyloid and tau proteins, and PET imaging (either metabolic or specifically for amyloid or tau; Albert et al., 2011). The utility of these biomarkers is increasingly well understood, albeit evolving. Clinical diagnosis typically utilizes bedside cognitive testing, commonly using the Mini-Mental Status Exam (MMSE). The MMSE has been examined in AD populations extensively, and using a cutoff of <24 for dementia results in fairly robust distinction between normal and AD dementia (AUC 0.85, PPV 97.0%, NPV 19.2%; Chapman et al., 2016). This provides robust detection of dementia, although performance is lower if looking for early disease states (manifesting as mild cognitive impairment) or if distinguishing between AD and other dementias. MRI can distinguish between normal vs. AD with sensitivity ranging from 33 to 98% and specificity ranging from 33 to 100% (Wollman and Prohovnik, 2003; Morinaga et al., 2010), with variability based on tradeoffs between sensitivity and specificity and improved with addition of quantitative volumetric analyses. A Cochrane Review of CSF tau based analyses found sensitivities range between 50 to >90% at specificities of <50 to >90% (median specificity of CSF total tau was 72%, and for CSF phosphorylated tau was 47.5%; Ritchie et al., 2017). PET imaging has the highest sensitivities and specificities of any testing modality, with sensitivities/specificities greater than 80%/86% for FDG PET and greater than 84%/86% for amyloid or tau specific imaging (AUC values, where reported, in the upper 80s to >90%). PET imaging also has demonstrated sensitivity to earlier states of disease and for distinguishing AD from non-AD dementia (Morinaga et al., 2010; Bloudek et al., 2011; La Joie, 2019; Lesman-Segev et al., 2021).

Sleep biomarkers do not play a role in AD diagnostics, and studies do not assess the diagnostic accuracy of sleep pathology for AD directly. Sleep might be thought to add little to the clinical management of AD beyond existing testing modalities. However, sleep may serve a critical role because of three factors. First, sleep pathology can be intervened upon with currently available therapies, potentially modifying disease risks. Lucey et al. (2023) administered suvorexant (which blocks the receptors for the wake promoting neurotransmitter, orexin) to healthy adults while monitoring CSF and found a 10% reduction in toxic soluble amyloid and tau species. These findings are driving an ongoing randomized clinical trial of suvorexant in AD patients (NCT04629547). Second, sleep is important for cognition in all people regardless of AD, and sleep pathology impairs attention, memory, and processing speed (Durmer, 2005; Alhola and Polo-Kantola, 2007; Csipo et al., 2021). Finally, sleep assessment (with EEG) is noninvasive, can be performed longitudinally in a patient’s home, and could be ubiquitously available with next generation EEG/sleep wearable technology at lower cost than any other biomarker listed. From publicly available data on hospital costs (legally required in the US by Hospital Price Transparency Regulations) we can find the costs of the above tests used clinically. Drawing from a sample of 9 large academic centers with memory disorders clinics drawn from the northeast, mid-atlantic, southeast, Midwest, south, and west coast who reported the costs of neurological testing (CPT code 78608 for brain PET, CPT code 70551 for MRI brain without contrast, CPT code 62270 for diagnostic lumbar puncture), we can get a rough estimate of the cost of each modality (note that costs vary wildly by geography and payor, so the lowest cash cost – not insurance adjusted rate - was used). The average cost of a PET scan (FDG PET, as no hospital listed the cost of amyloid or tau specific imaging) was $2,411, MRI brain without contrast was $966, and lumbar puncture was $777 (not including the actual amyloid and tau testing, which only 3 hospitals listed and cost an additional $400 to $1144). In contrast, the average cost of a home sleep study was $338 for the 8 hospitals providing this charge (CPT code 96806, although this code does not typically include EEG as this is not typically available as an at-home test due to the limited hardware options). The combination of these factors makes sleep a potentially valuable area of research in AD. Furthermore, future multivariate mixed models could provide substantially greater insight into AD using sleep macro-and micro-architectural features that can be measured with objectively.

## Implications and future directions

While sleep disturbances are common in a variety of neuropsychiatric diseases, the causative versus associative relationships between sleep and neurodegeneration require further study. Nevertheless, the importance of sleep in the development, progression, and symptom expression of neurodegenerative diseases is coming to light and already has shown promise as a potential biomarker of disease progression in AD. One major limitation is the ability to capture adequate sleep data. Traditional polysomnography (PSG), the gold standard for monitoring brain activity in sleep, is expensive, requires significant equipment and personnel resources, and has limited availability—all of which preclude using PSG as a longitudinal or periodic measure of sleep parameters. Actigraphy and watch-based sleep monitoring using accelerometry and pulse data are low-cost and non-invasive approaches, but may inaccurately portray sleep metrics and do not measure brain activity. Nevertheless, wearable technology, especially with addition of EEG, offers the possibility of longitudinal recording over long time periods to observe changes in brain function with age (Ye et al., 2020; though the value of 24-h or more monitoring has not yet been justified; Lysen et al., 2020).

The future of sleep-based biomarkers relies on technical advances in simplified, reliable, and reproducible sensors that include EEG data collection in the home setting and are comfortable enough for repeated usage. This level of data is required to understand the macro-and micro-architectural features found to be related to AD and normal aging. As more neurophysiological data becomes available from neurodegenerative diseases, new opportunities will arise for sleep physiology-based models serving as risk and treatment biomarkers that can be implemented for various contexts of use, including clinical research and drug development (such as prognostic, patient stratification, predictive or target engagement as pharmacodynamic markers). Sleep is a critical component of life that matters to patients, providers, and regulators. Sleep offers both a window into brain function and dysfunction and an opportune target for clinical research and drug development.
